# Recapitulating T cell infiltration in 3D psoriatic skin models for patient-specific drug testing

**DOI:** 10.1038/s41598-020-60275-0

**Published:** 2020-03-05

**Authors:** Jung U Shin, Hasan E. Abaci, Lauren Herron, Zongyou Guo, Brigitte Sallee, Alberto Pappalardo, Joanna Jackow, Eddy Hsi Chun Wang, Yanne Doucet, Angela M. Christiano

**Affiliations:** 1 Department of Dermatology, Columbia University Medical Center, NY New York, USA; 2 Department of Dermatology, CHA Bundang Medical Center, CHA University, Seongnam, Republic of Korea; 3 Department of Genetics & Development, Columbia University, New York, NY USA

**Keywords:** Skin diseases, Biomedical engineering

## Abstract

Drug screening studies for inflammatory skin diseases are currently performed using model systems that only partially recapitulate human diseased skin. Here, we developed a new strategy to incorporate T cells into human 3D skin constructs (HSCs), which enabled us to closely monitor and quantitate T cell responses. We found that the epidermis promotes the activation and infiltration of T cells into the skin, and provides a directional cue for their selective migration towards the epidermis. We established a psoriatic HSC (pHSC) by incorporating polarized Th1/Th17 cells or CCR6+CLA+ T cells derived from psoriasis patients into the constructs. These pHSCs showed a psoriatic epidermal phenotype and characteristic cytokine profiles, and responded to various classes of psoriasis drugs, highlighting the potential utility of our model as a drug screening platform. Taken together, we developed an advanced immunocompetent 3D skin model to investigate epidermal-T cell interactions and to understand the pathophysiology of inflammatory skin diseases in a human-relevant and patient-specific context.

## Introduction

Skin is equipped with a complex immune system that involves various immune cell types, such as T cells, Langerhans cells, dermal dendritic cells, mast cells, and basophils, which secrete cytokines and chemokines to regulate immune responses both locally and systemically. Perturbations in this complex regulatory mechanism due to genetic and/or environmental factors contribute to the development of inflammatory skin diseases^1^. Therefore, understanding the skin immune system is essential to investigate the pathogenesis of inflammatory skin diseases and to discover and validate effective targets for treatment.

Psoriasis affects 2–3% of the Western population^2,3^ and is a chronic inflammatory skin disease with dysregulated keratinocyte proliferation^4^. In the pathogenesis of psoriasis, the interplay between keratinocytes and immune cells is important for the initiation, progression and persistence of the disease. Psoriatic keratinocytes recruit and activate plasmacytoid dendritic cells and neutrophils to initiate psoriasis^5–8^, and produce autoantigens, such as LL37^5–7^, ADAMTSL5^9^, and PLA2G4D^10^ to activate T cells. Once T cells and DCs are recruited and activated, a cytokine niche is established including IFNs, IL-17, TNFα, and IL-22^11–13^. These cytokines not only alter epidermal proliferation and differentiation^12,13^, but also activate keratinocytes to release chemokines and chemoattractants, which induce further recruitment and infiltration of inflammatory cells into the skin^11^. Although this complex feedback loop between keratinocytes and immune cells is central in the pathogenesis of psoriasis, current *in vitro* models do not capture these cellular interactions, such as migration of the immune cells, highlighting the need for an advanced model that recapitulates the physiological and immunological complexity of the disease.

Although there have been improvements in the efficacy of biologic therapies, therapeutic outcomes vary among patients, and there is no reliable model to predict individual efficacy prior to treatment. There are several psoriasis mouse models and 2D cell culture models, however these do not fully represent human pathophysiology or enable prediction of patient-specific responses. To overcome these limitations, engineered human skin constructs (HSCs) have been utilized to model psoriasis. Most of the previous HSC-based psoriasis models were limited to those composed of patient-derived keratinocytes (KCs) or fibroblasts (FBs), or those using wild-type KCs and FBs treated with psoriasis-related cytokines^14–19^, however, these models lacked immune cells and did not recapitulate disease physiology. One study^20^ induced a psoriasiform skin phenotype by using *in vitro* polarized T cells to repopulate decellularized skin with normal fibroblasts and keratinocytes. However, the incorporation of human disease- or patient-specific T cells into HSCs to recapitulate a clinically-relevant disease phenotype has not been accomplished.

Recent work from our group and others included the incorporation of many important skin components such as melanocytes, hair follicles, and vasculature into HSCs^21–24^. Here, we developed a bioengineering method to incorporate immune cells into HSCs to capture their migration and interaction with the epidermis. We developed a human-relevant *in vitro* model of psoriasis incorporating patient-specific immune cells in HSCs (pHSCs). We validated our model pharmacologically using multiple classes of psoriasis drugs including conventional corticosteroids, cytokine neutralizing antibodies and phosphodiesterase (PDE) 4 inhibitors. Our study establishes an advanced approach to recapitulate inflammatory skin diseases using patient-specific cells and a physiological *in vitro* platform that allows for dissecting epidermal and immune cell interactions as well as quantification of T cell migration into the skin in the context of disease progression and drug treatment.

## Results

### Infiltration of T cells into the skin

**Figure 1:**
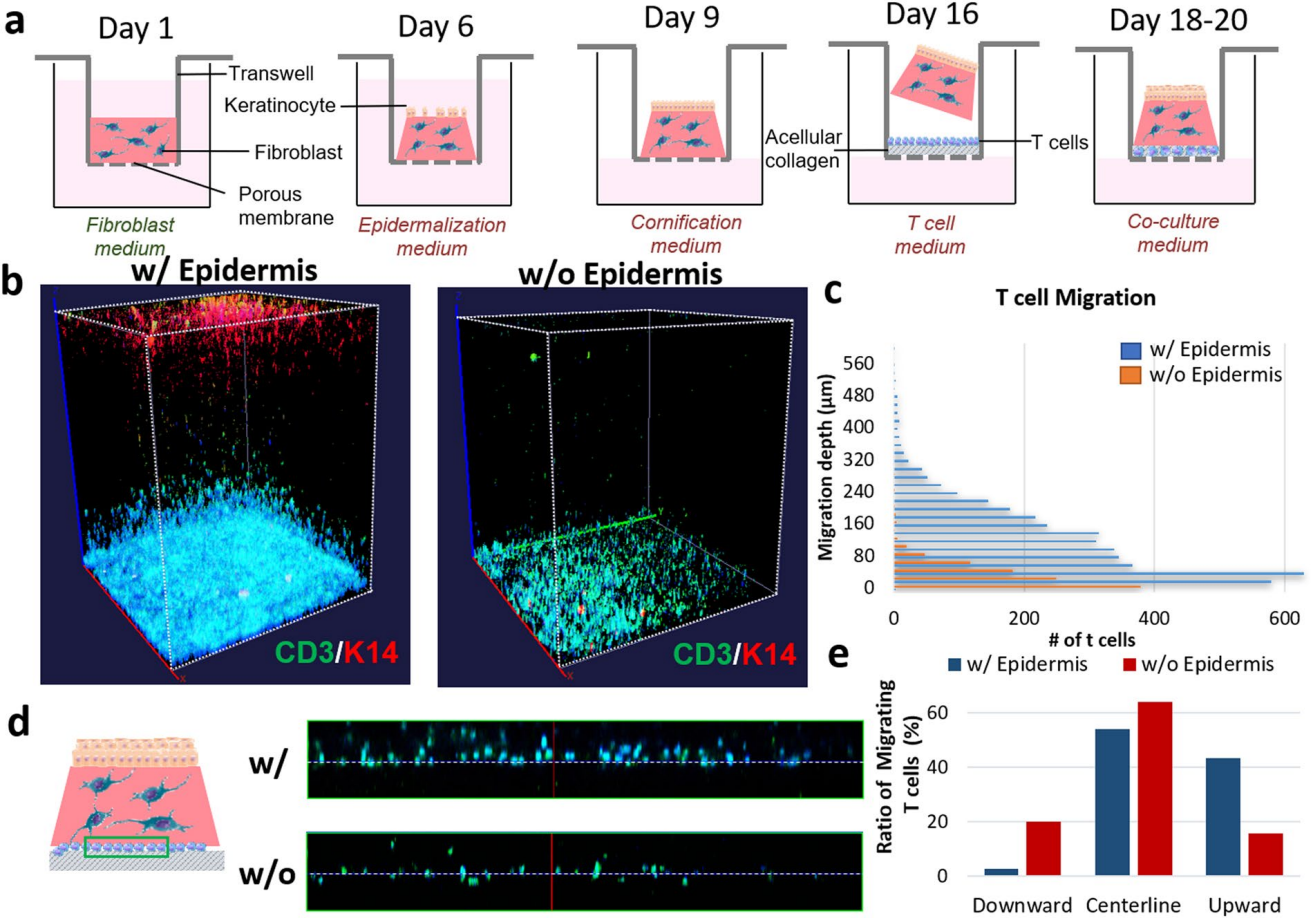
Inducing the infiltration of CD4+ T cells into HSCs. **(a)** Method for generation of immunocompetent HSC. **(b)** 3D-reconstructed whole-mount image of HSCs showing 3D conformation of K14-positive epidermis and CD3-positive T cells with and without the epidermis (DAPI: blue). **(c)** Quantification of the number and penetration depth of infiltrated T cells in HSCs (µm). **(d)** Orthogonal segment of T cell-bearing HSCs with the centerline of their initial position on the gel surface as a reference (white dotted line) showing CD3-positive (green) T cells (DAPI: blue). **(e)** Quantification of the total number of cells that migrated upward (dermis) or downward (acellular gel).

As part of the pathological immune response in human skin, circulating T cells infiltrate into the skin and migrate toward the epidermis through chemotactic signals from epidermal cells. To recapitulate this process, we integrated CD4+ T cells onto the bottom surface of engineered HSCs and monitored their migratory behavior in the dermis. We first generated HSCs that are composed of dermal fibroblasts embedded in a collagen type I gel and keratinocytes in a transwell culture system at the air-liquid interface^24^ (Fig. 1a). Following the formation of a fully-differentiated epidermis, we prepared a thin, acellular layer of collagen gel in a separate transwell insert and seeded CD4+ T cells that were activated with anti-CD3 and anti-CD28 on top. After activation, T cells attached on the acellular gel overnight where they cover the gel surface (Supplementary Fig. 1a). Subsequently, we transferred HSCs onto the T cells, and co-cultured them in a common medium (see Methods) for 4 days. T cells migrated into the dermis and retained their proliferative state (Supplementary Fig. 1b,c).

To determine the effect of the epidermis on T cell migration, in one set of constructs we mechanically removed the epidermis prior to the experiment. The constructs with the epidermis exhibited significantly higher numbers of infiltrating T cells at every layer in the dermis and deeper penetration toward the epidermis, compared to HSCs without the epidermis (Fig. 1b,c). In HSCs with the epidermis intact, significant numbers of migrating T cells reached a penetration distance up to 500 µm into the dermis, whereas no significant numbers of T cells were detected above 100 µm in HSCs without the epidermis (Fig. 1c). To quantitate the T cells that migrated in the direction of the epidermis, we counted the total number of cells that moved upward (dermis) or downward (acellular gel) taking the centerline demarcating their initial position on the gel surface as a reference. We found that in HSCs with the epidermis, there were significantly more T cells migrated in the upward direction (toward the epidermis) compared to downward, whereas in the HSC without the epidermis, there was no significant difference in the direction of migration (Fig. 1d,e). Taken together, this data suggested that the epidermis promoted CD4+ T cell migration, generating a unidirectional migratory response.

**Figure 2:**
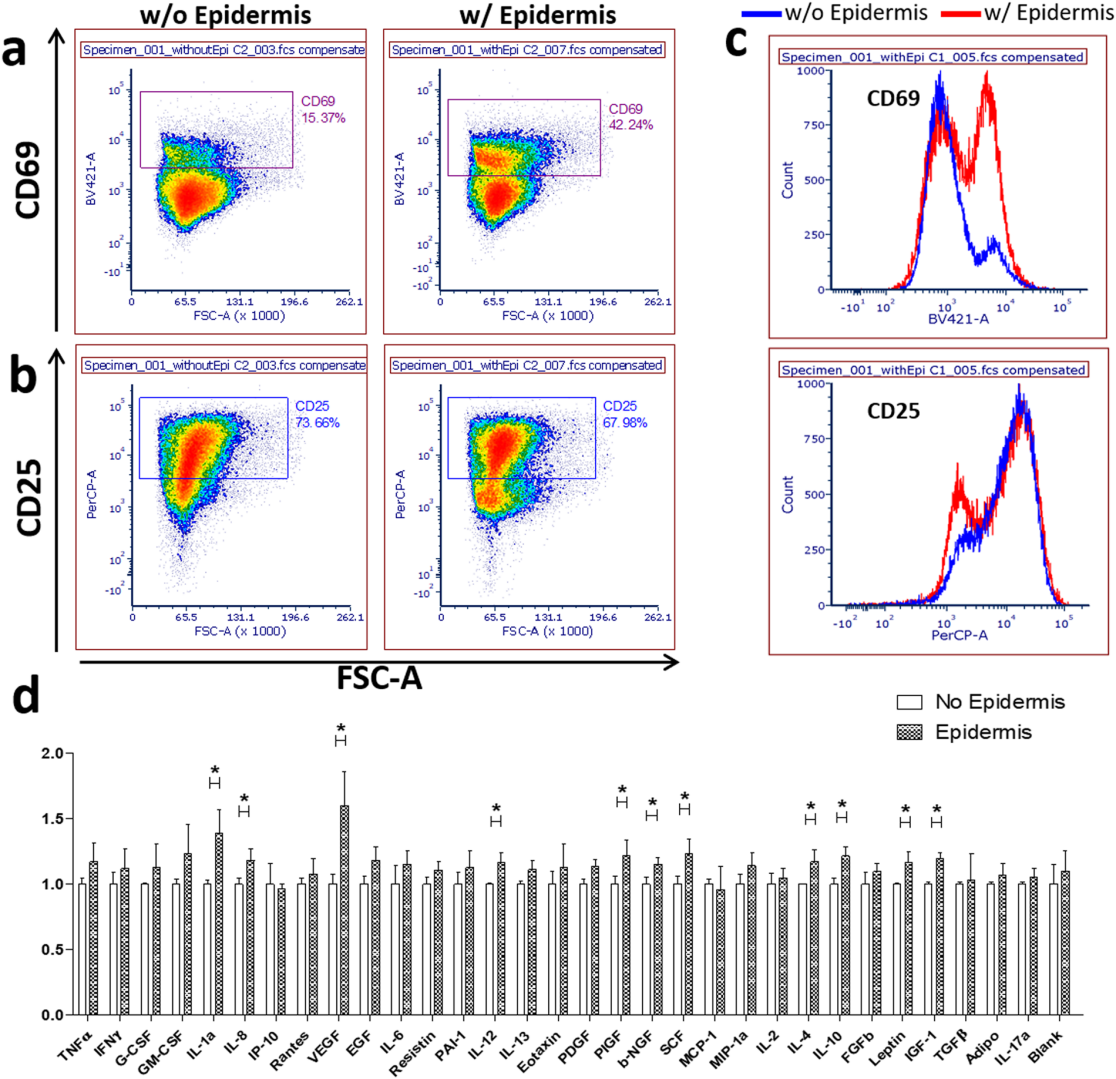
The effect of the epidermis on CD4+ T cell activation. Immunofluorescent staining and flow cytometry analysis of T cell activation markers, CD69 **(a)** and CD25 **(b)**, in CD3+ T cells isolated from HSCs with the epidermis or without the epidermis **(b)**. **(c)** Histograms for CD69 (upper panel) and CD25 (lower panel) on CD3+ T cells from HSCs with the epidermis (red) or HSCs without the epidermis (blue). **(d)** Cytokine secretion from T cell-bearing HSCs with or without the epidermis using cytokine ELISA plate arrays (t-test; *p < 0.01, N = 3).

We further examined the effects of the epidermis on T cell activation by isolating the T cells that migrated into the dermis in HSCs with or without the epidermis and performed flow cytometry for surface activation markers, CD25 and CD69. In HSCs with the epidermis, 42% of T cells that migrated into the dermis expressed CD69 (Fig. 2a), whereas in HSCs without the epidermis, only 15% of T cells showed CD69 expression (Fig. 2b), suggesting that the epidermis participates in the activation of T cells. There was no significant difference in the expression of CD25 between the two conditions (Fig. 2a–c). Furthermore, HSCs with the epidermis released more cytokines, such as IL-1a, IL-4, IL-8, IL-10, IL-12, and SCF as measured by ELISA (Fig. 2d), indicating that epidermis-T cell interactions can activate inflammation and recruit immune cells into the skin. Along with cytokines, several growth factors, such as VEGF, PIGF, b-NGF, IGF-1, and leptin were increased in HSCs with the epidermis, suggesting a potential role of epidermis-T cell interactions in the growth, proliferation, and differentiation of other cell types, such as endothelial cells, fibroblasts, neurons, and adipocytes.

### Establishing the psoriatic phenotype using *in vitro*-polarized T cells

**Figure 3:**
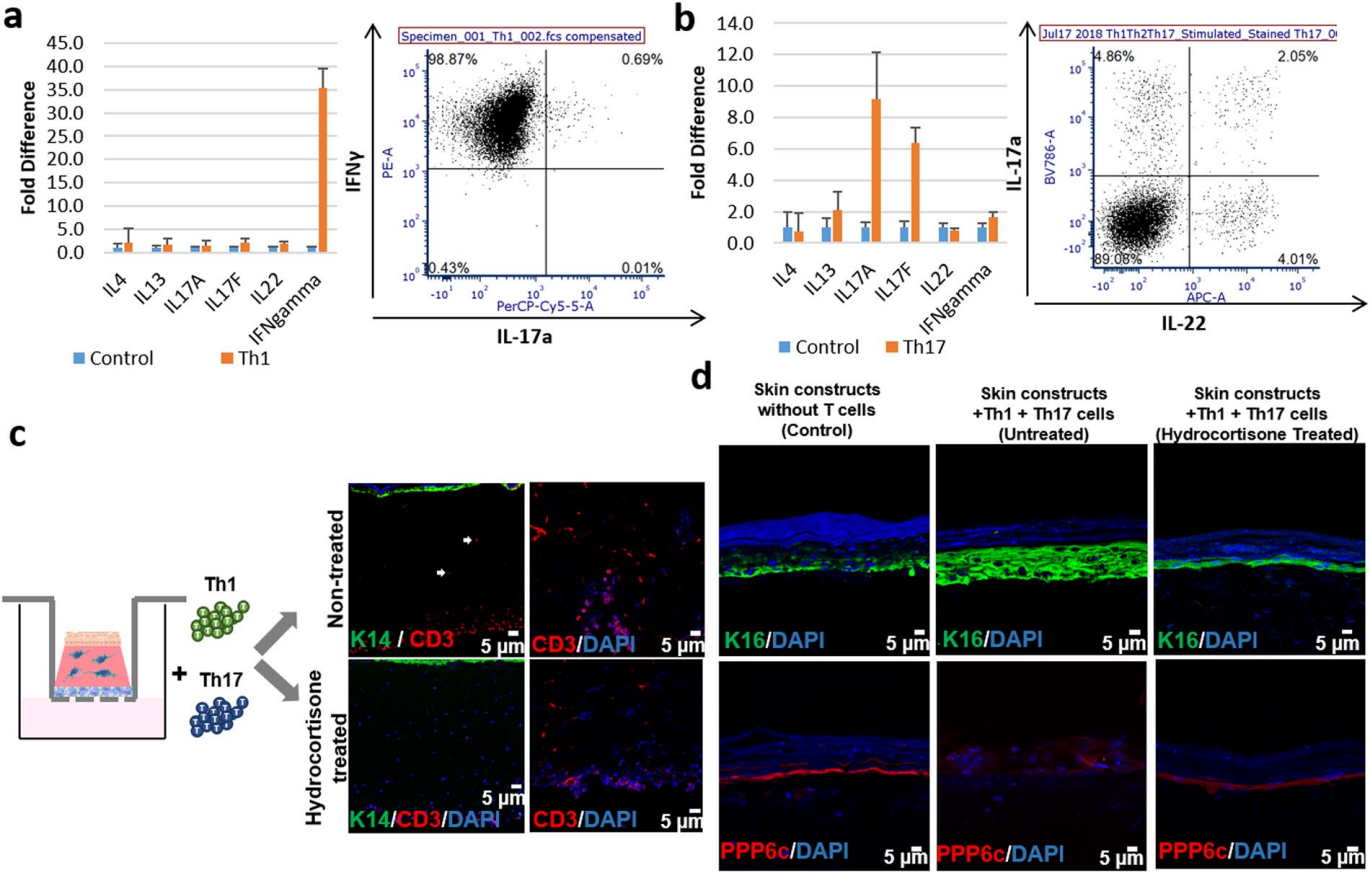
Change of the epidermal phenotype and T cell migration in response to hydrocortisone in Th1/Th17 incorporated HSCs. Cytokine expression analyzed by qRT-PCR and flow cytometry analysis of *in vitro* polarized Th1 cells (**a**) and Th17 cells (**b**). (**c**) Immunofluorescence staining of K14 (green) and CD3 (red) in Th1/Th17-bearing HSCs with or without hydrocortisone treatment. (**d**) Immunofluorescence staining of K16 (green) and PPP6c (red) in Th1/Th17-bearing HSCs with or without hydrocortisone treatment.

After validating the effect of the epidermis on T cell migration and activation in HSCs, we next examined whether the incorporation of disease-relevant T cells could induce a psoriatic epidermal phenotype. For this purpose, we first polarized Th1 and Th17 cells according to previous protocols^25,26^. Cytokine production from *in vitro* polarized Th1 and Th17 cells was evaluated using qRT-PCR and flow cytometry analysis. *In vitro* polarized Th1 cells showed higher IFNγ gene expression than non-polarized cells. We found that 95.37 ± 3.77% of the Th1 polarized cells were positively stained with anti-IFNγ antibody (Fig. 3a), compared to 13.66 ± 3.61% in the non-polarized cells. Likewise, Th17 polarized cells showed higher IL-17A and IL-17F gene expression than non-polarized cells, where 6.7 ± 0.2% of Th17 polarized cells positively stained with anti-IL-17a antibody (Fig. 3b), compared to 0.71 ± 0.57% of non-polarized cells.

Using the method that we described in Fig. 1a, we incorporated polarized Th1 and Th17 cells into the fully differentiated HSCs to build pHSCs. Th1 and Th17 cells successfully migrated into HSCs four days after their placement underneath HSCs (Fig. 3c). Th1/Th17-bearing pHSCs exhibited a higher expression of K16, a lower expression of PPP6c, and thicker epidermis (Fig. 3d and Supplementary Fig. 2), resembling the psoriatic epidermal phenotype^27,28^.

We further evaluated whether our Th1/Th17-bearing pHSCs were responsive to psoriasis drugs. Hydrocortisone, a broad-spectrum immunosuppressant, is well-known to downregulate T cell function^29^. We treated our Th1/Th17-bearing-pHSCs with hydrocortisone to evaluate whether it modulated T cell infiltration and the psoriatic phenotype. Hydrocortisone treatment significantly slowed T cell migration, and T cells were retained between acellular collagen base and dermal part of the skin constructs (Fig. 3c). Moreover, hydrocortisone treatment inhibited the overexpression of K16 and the downregulation of PPP6c (Fig. 3d).

**Figure 4:**
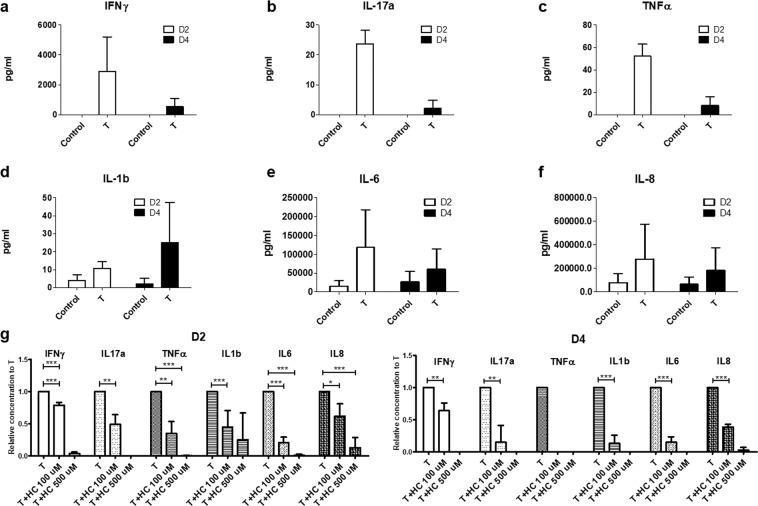
Change of cytokine profile in Th1/Th17-bearing HSCs. **(a–f)** Cytokine secretion measured by ELISA over four days in two day intervals from Th1/Th17-bearing HSCs; **(a)** IFNγ, **(b)** IL-17a, **(c)** TNFα, **(d)** IL-1b, **(e)** IL-6, and **(f)** IL-8. **(g)** Relative cytokine secretion of IFNγ, IL-17a, TNFα, IL-1b, IL-6, and IL-8 from Th1/Th17-bearing HSCs after 100 μM and 500 μM of hydrocortisone treatment on day 2 and day 4.

Next, we evaluated whether Th1/Th17 cells continued secreting psoriasis-relevant cytokines after their incorporation into HSCs. T cell-secreted cytokines, IFNγ, IL-17a and TNFα, were not detected in the supernatant of HSCs without T cells. In contrast, Th1/Th17-bearing pHSCs still produced IFNγ, IL-17a and TNFα. These cytokines were mainly secreted within the first 2 days (D2) and decreased in the following 2 days (D4) (Fig. 4a–c). To determine the effect of Th1/Th17 incorporation on keratinocytes in the HSCs, we evaluated the concentration of pro-inflammatory cytokines secreted by keratinocytes. We found that Th1/Th17-bearing pHSCs produced higher concentrations of pro-inflammatory cytokines, IL-1b, IL-6, and IL-8, suggesting that Th1/Th17 cells induced the upregulation of pro-inflammatory cytokines in keratinocytes (Fig. 4d–f). To further understand drug responsiveness of cytokine production, we evaluated the change of cytokine concentration in the supernatant after 100 μM and 500 μM of hydrocortisone treatment. We found that hydrocortisone treatment significantly decreased cytokine secretion in Th1/Th17-bearing pHSCs in a dose dependent manner (Fig. 4g). Finally, to test whether the profile of cytokine expression in our constructs was specific to the psoriatic phenotype, we evaluated whether our model secreted any atopic dermatitis-related cytokines, such as TSLP and TARC, using ELISA, and found no evidence of increased cytokine levels (Supplementary Fig. 3a). Accordingly, we did not see a corresponding significant upregulation of the expression of TSLP or TARC by qRT-PCR in our pHSCs (Supplementary Fig. 3b).

### Normalization of the psoriasiform phenotype using a PDE4 inhibitor and anti-IL17a antibody

To further validate Th1/Th17-bearing pHSCs as a psoriatic inflammation model, we examined whether the psoriatic phenotype in our HSC model could be reversed by other classes of drugs, such as recently discovered small molecules and cytokine targeting drugs, which are used clinically to inhibit specific targets involved in disease etiology. Phosphodiesterase 4 (PDE4) is an enzyme that degrades cAMP, which is the secondary messenger regulating the production of proinflammatory cytokines^30^. Inhibition of PDE4 leads to decreased production of inflammatory mediators in both peripheral blood mononuclear cells (PBMCs) and keratinocytes^31^, and is clinically effective in treatment of psoriasis^30^. IL-17a is a prominent cytokine in psoriasis pathogenesis, and its inhibition with neutralizing antibody recently showed successful outcome in treating patients^32^. Therefore, we treated our Th1/Th17–bearing pHSCs with 2.5 μM PDE4 inhibitor or 0.1 μg/ml neutralizing anti-IL17a antibody.

**Figure 5:**
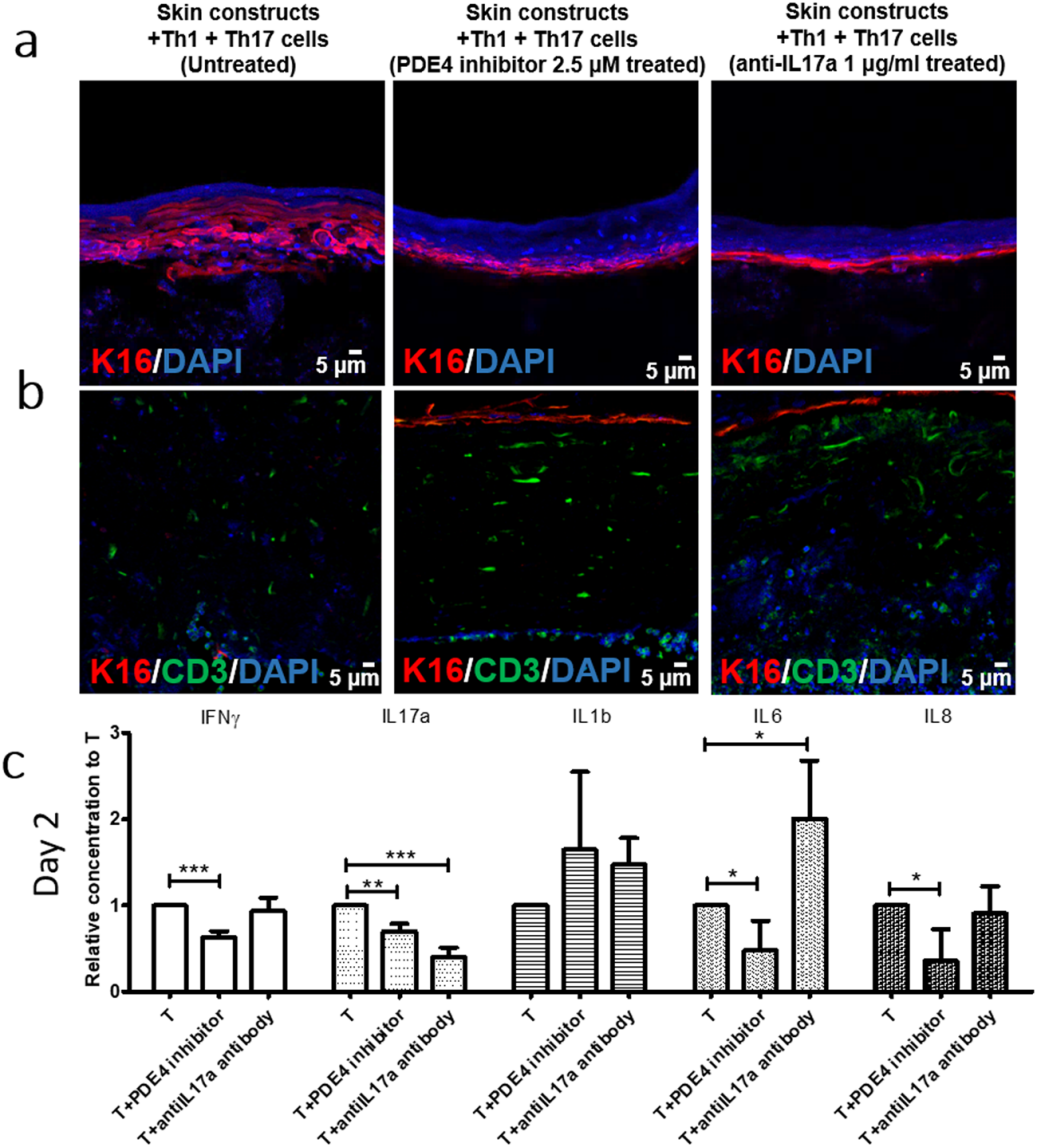
Effect of PDE4 inhibitor and anti-IL17a antibody on Th1/Th17-incorporated 3D skin. Immunofluorescence staining of **(a)** K16 (red) and **(b)** CD3 (green) in Th1/Th17-bearing HSCs without treatment (left panel), with 2.5 μM PDE4 inhibitor treatment (middle panel), and 1 μg/ml anti-IL17a antibody treatment (left panel). **(c)** Relative cytokine secretion of IFNγ, IL-17a, IL-1b, IL-6, and IL-8 from Th1/Th17-bearing HSCs after PDE4 inhibitor or anti-IL17a antibody treatment on day 2.

Using PDE4 inhibitor treatment, both K16 expression in the epidermis and T cell infiltration were inhibited (Fig. 5a). Interestingly, although anti-IL17a antibody reversed K16 expression in the epidermis, it did not inhibit T cell migration (Fig. 5b), consistent with previous human data, in which considerable numbers of CD3+ T cells remained in the skin after blockade of IL-17^33^. Similarly, in another human clinical trial, blockade of IL-17R signaling generated a rapid reduction in inflammatory keratinocyte gene expression, whereas this effect was delayed for infiltrating leukocytes^34^. Regarding the effect of anti-IL17a antibody on cytokine production, anti-IL17a antibody reduced the concentration of IL-17a more specifically than the PDE4 inhibitor. In pHSCs treated with the anti-IL17a antibody, IL-17a concentration was significantly decreased, whereas the concentration of IFNγ, IL-1b or IL-8 was not affected. IL-6 concentration was increased with anti-IL17a antibody treatment, whereas the PDE4 inhibitor showed broader inhibitory effects on cytokine secretion compared to anti-IL17a antibody. The PDE4 inhibitor-treated pHSCs showed decreased secretion of IFNγ, IL-17a, IL-6, and IL-8 (Fig. 5c and Supplementary Fig. 4).

### Developing psoriatic HSCs using patient-specific T cells

**Figure 6:**
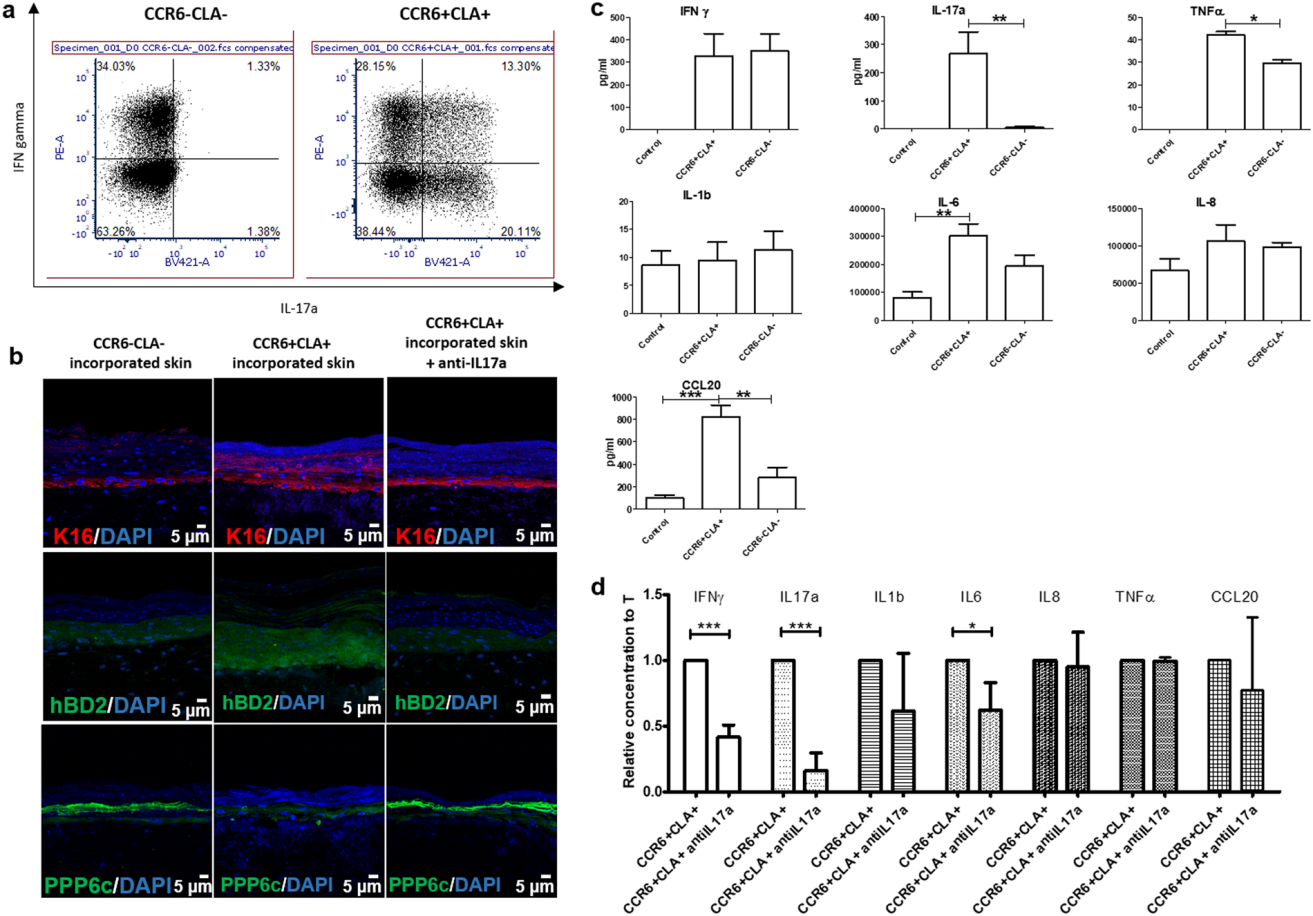
Epidermal phenotype and cytokine profile of HSCs containing patient-derived T cells. **(a)** Flow cytometry analysis for intracellular IFNa and IL-17a from patient-derived CCR6+CLA+ skin homing T cells and CCR6−CLA- T cells. **(b)** Immunofluorescence staining of K16 (red), hBD2 (green), and PPP6c (green) in CCR6−CLA- T cell-bearing HSCs (left panel), CCR6+CLA+ T cell-bearing HSCs (middle panel), and CCR6+CLA+ T cell-bearing HSCs treated with anti-IL17a antibody (right panel). **(c)** Cytokine secretion measured by ELISA over two days in CCR6−CLA- T cell-bearing HSCs, and CCR6+CLA+ T cell-bearing HSCs. **(d)** Relative cytokine secretion over two days in CCR6+CLA+ T cell-bearing HSCs, and CCR6+CLA+ T cell-bearing HSCs treated with anti-IL17a antibody.

Cutaneous lymphocyte antigen (CLA), a marker of skin homing T cells, is expressed on infiltrating T cells in psoriatic lesions^35^, and its expression is correlated with disease severity of psoriasis^36^. The chemokine receptor CCR6 is a marker for T cells secreting IL-17 and IL-22, which are the key cytokines in psoriasis pathogenesis^37–39^. To determine whether patient-derived T cells can induce psoriatic inflammation, similar to *in vitro* polarized Th1/Th17 cells, we isolated CCR6− and CLA− T cells (CCR6−CLA−) and CCR6+ or CLA+ T cells (CCR6+CLA+), and incorporated them into HSCs to evaluate the epidermal phenotype and cytokine profile. Isolated CCR6+CLA+ T cells showed higher IFNγ and IL-17a production compared to CCR6−CLA− T cells (Fig. 6a).

CCR6+CLA+ T cell-bearing pHSCs showed increased K16 and hBD2, and decreased PPP6c, representing the psoriatic epidermal phenotype. Treatment with neutralizing anti-IL17a antibody reversed these markers, indicating drug responsiveness of patient-derived T cell-incorporated pHSCs (Fig. 6b). CCR6+CLA+ T cell-bearing pHSCs produced higher IL-17a, TNFα, IL-6, and CCL20 compared to HSCs without T cells or with CCR6−CLA− T cells (Fig. 6c). Interestingly, anti-IL17a antibody treatment of CCR6+CLA+ T cell-bearing pHSCs demonstrated decreased production of not only IL-17a, but also IFNγ and IL-6, whereas same treatment only reduced IL-17a in polarized Th1/Th17 cell-bearing pHSCs, suggesting a difference between patient-derived T cell-bearing pHSCs and polarized Th1/Th17 cell-bearing pHSCs (Fig. 6d).

## Discussion

The pathogenesis of many diseases including infectious, autoimmune, allergic, and other inflammatory diseases is driven by immune reactions against pathogens, autoantigens, allergens or perturbations in the immune system. Therefore, there is a growing interest in the development of engineered human immune tissues, such as skin, lymph node, thymus, and bone marrow, for more accurate recapitulation of human immunity^40^. In our study, we developed an engineering strategy to incorporate disease- and patient- specific immune components into HSCs to model psoriasis in a human-relevant context, which enables studying epidermis-T cell interactions and quantitating the migration dynamics of T cells into the skin, neither of which was possible using previous models. We validated our model as a drug testing platform using polarized Th1/Th17 cells and patient-specific cells, and showed that it is pharmacologically responsive, thereby representing a major step towards individual drug screening for psoriasis patients.

Psoriasis is a chronic inflammatory skin disease with complex pathophysiology attributed to various genetic and environmental factors. Although recent developments and success in the use of biologic therapies highlighted that immune regulation plays a vital role in the development of psoriasis, there is also cumulative evidence that interaction of epidermal factors and the immune system play a synergistic role in the initiation and maintenance of disease. Since clinical studies have practical and ethical limitations, conventional 2D culture and animal models have been widely utilized^41–47^. A large body of previous research focused on developing 3D *in vitro* psoriasis models, which can mimic psoriasis pathophysiology^14–19,48,49^. Currently, 3D skin constructs generated from healthy keratinocytes and psoriasis patient-derived fibroblasts are commercially available from vendors. Although previous models can recapitulate some aspects of psoriatic inflammation, the lack of a complex immunologic milieu and limited availability of patient-derived cells limits their utility.

For better recapitulation of the complex tissue microenvironment, previous studies^20^ incorporated T cells into full-thickness skin constructs made of decellularized dermis repopulated with healthy dermal fibroblasts and keratinocytes. Incorporation of activated or *in vitro*-polarized T cells led to a psoriatic phenotype of the epidermis along with cytokine and chemokine alterations. Building upon these previous models, here we developed a method to incorporate T cells into the HSCs, and confirmed its capabilities to evaluate the migration of T cells and drug responsiveness. Our method enabled quantification of the depth and direction of T cell migration and to evaluate the activation of skin-infiltrated T cells in the presence and absence of the epidermis.

We demonstrated that the presence of the epidermis is a critical factor providing directional cues to T cells to selectively infiltrate toward the epidermis, mimicking physiological T cell infiltration under inflammatory conditions. In addition to the migratory behavior of T cells, the presence of the epidermis induced the T cell activation marker, CD69, suggesting that interactions between T cells and keratinocytes may play a role in T cell activation in the local tissue. Although both CD25 and CD69 are T cell activation markers, they have different kinetics. The expression of CD25 can persist for 7 days after T-cell receptor (TCR) ligation, whereas the expression of CD69 usually diminishes 48-72 hours after activation. Based on our observations of prolonged CD69 expression in HSCs with the epidermis, we postulated that the epidermis is involved in the maintenance of CD69 upregulation. Then, we sought to define the cytokine activity to explain polarized migration of T cells and prolonged expression of CD69. We found that IL-1a, IL-8, VEGF, IL-12, PIGF, b-NGF, SCF, IL-4, IL-10, Leptin, and IGF-1 levels were significantly increased in HSCs with the epidermis compared to those without the epidermis, suggesting that altered cytokines and chemokines secreted by the epidermis could induce T cell migratory behavior and increased CD69 expression. Further investigation will determine which cytokines or chemokines are responsible for the phenotypic and functional changes of T cells in immunocompetent 3D skin models.

In addition, we established CCR6+CLA+ T cell-bearing pHSCs with inducible and reversible psoriatic inflammation comparable to that achieved with polarized Th1/Th17 model. Interestingly, anti-IL17a antibody treatment reduced IFNγ, IL-17a, and IL-6 production in CCR6+CLA+ T cell-bearing pHSCs, consistent with the decreased T cell associated cytokines after IL-17 blockade in human clinical trial^34^. However, same treatment reduced only IL-17a in the polarized Th1/Th17 model. This may be explained by extremely high expression of IFNγ from polarized Th1/Th17 models compared to CCR6+CLA+ T cell-bearing pHSCs, suggesting that selection of an appropriate cell population is critical to predict biologically relevant drug responses. Incorporation of patient-derived skin-homing T cells in our model can be used in the future for predicting the response of patients to treatments and accelerate the development of personalized medicine.

Although our platform represents a significant step forward in modeling inflammatory skin diseases in HSCs, it can still be refined in the future. Other psoriasis-related immune cells, such as neutrophils, dendritic cells, and macrophages can help to improve the immunological relevance of our model to fully recapitulate psoriatic inflammation in the skin. Since our model only includes CD4+ T cells, but not CD8+ T cells or dendritic cells, we expect any unintended immune reactions arising from the donor mismatch between keratinocytes and T cells to be minimal. However, to apply this approach for other autoimmune diseases and graft-versus-host disease, it will be necessary to consider using donor-matched keratinocytes, fibroblasts, and immune cells. In that case, iPSC-derived skin cells will be an important alternative to address practical difficulties in obtaining multiple tissue types from the same donor^23,50,51^. In addition, considering that other skin components, such as hair follicles^52^, vasculature^24^ and adipose tissue^53^ also play a role in the regulation of immune reactions in the skin, these components can be included in future constructs using recently developed tissue engineering approaches by our group^21,24^ and others^54^.

This study provides an advanced method to incorporate patient-derived T cells into HSCs to faithfully recapitulate immune responses in the skin. The ability to monitor and quantitate T cell infiltration into the HSCs during psoriatic inflammation, as well as in response to drug treatment, represents an invaluable new read-out for preclinical drug testing studies. Moreover, the response of patient-derived T cells in our model to anti-IL17a antibody treatment invites the application of this model for individualized drug screening. This strategy can be scaled and widely adapted for modeling other inflammatory skin diseases, such as atopic dermatitis, vitiligo, allergic contact dermatitis, or alopecia areata. Integrating our model with the state-of-the-art technologies, such as 3D bioprinting or skin-on-a-chip platforms^55^, will allow the expansion of this model for applications, such as high-throughput drug screening and to investigate systemic consequences of inflammatory skin diseases using multi-tissue chips.

## Materials and Methods

### Human samples

Human blood samples were obtained from 3 healthy controls (HCs) and from 4 psoriasis patients. The patients did not receive any systemic or topical treatment with immunosuppressive drugs for at least 4 weeks before blood sample collection. This study was approved by an Institutional Review Board at Columbia University (AAAI0706), and was performed in accordance with relevant guidelines/regulations. Informed consent was obtained from all subjects before they participated in the study.

Epidermal keratinocytes and fibroblasts were isolated from neonatal foreskin from healthy individuals from the Children’s Hospital at Columbia University Medical Center. Samples were de-identified and designated as nonhuman subject research under 45 CFR Part 46, under Institutional Review Board exemption at Columbia University.

### Keratinocyte and fibroblast culture

Normal epidermal keratinocytes were isolated and cultured in CnT-07 media (CELLnTEC). Normal dermal fibroblasts were isolated in 0.3% collagenase and cultured in fibroblast medium (DMEM culture media (Life Technologies) supplemented with 10% fetal bovine serum (FBS) (Life Technologies) and 1% penicillin-streptomycin (Life Technologies)). Cells were incubated at 37 °C in a 5% CO_2_ atmosphere and media were replenished every second or third day.

### Generation of immunocompetent 3D skin equivalents

3D skin constructs were generated by adapting a previously described protocol^25^. Briefly, a type I collagen matrix (containing 0.5 ×10^6^ fibroblasts) was deposited onto polyethylene terephthalate membranes (BD Biosciences), and allowed to polymerize. After incubation of the polymerized matrix for 7 days, 1 ×10^6^ keratinocytes were seeded onto the matrix, and incubated for a further 7 days. The composite culture was raised to the air-liquid interface and fed from below to induce epidermal differentiation. 3D skin constructs were harvested 14 days later and either snap frozen in LN2 or embedded in wax. For immunocompetent 3D skin, 1 ×10^6^ T cells were cultured overnight on collagen gel and fully differentiated 3D skin constructs were transferred onto the T cell attached collagen gel. Immunocompetent 3D skin was cultured in 1:1 10% FBS supplemented advanced RPMI (Invitrogen) medium and skin medium and supplemented with 10U or 30U of IL-2 (Roche).

### Drugs and chemicals

Anti-Human IL-17A mAb (MT504, Mabtech), hydrocortisone (H0888, Sigma-Aldrich), apremilast (AdooQBioScience) were used in concentrations as indicated in the figure legends.

### *In vitro* culture and polarization of T cells into Th1 and Th17 cells

*In vitro* Th1 polarization was performed using a modified protocol^25,26^. Naive CD4+ T cells were purified from PBMCs by negative selection using MACS separation according to the manufacturer’s instructions (Miltenyi Biotec, Sunnyvale, CA) and were cultured at 37 °C in 5% CO_2_ in advanced RPMI media (Invitrogen) supplemented with 10% heat inactivated FBS (Gibco). Naive cells (0.5 ×10^6^/ml) were plated in 24-well plates with beads coated with anti-CD3 and anti-CD28 (Dynabeads; Invitrogen Dynal, Oslo, Norway) at a concentration of one bead per cell. Abs and cytokines were added at the time of plating at the following concentrations: 20 ng/ml IL-12 (R&D), 5 µg/ml anti–IL-4 (eBioscience). On day 3 of polarization, 30 U/ml IL-2 (Roche) was added. *In vitro* Th17 polarization was performed using a modified protocol from Manel *et al*.^56^ and Keerthivasan *et al*.^57^. Naive cells (0.33 ×10^6^/ml) were plated in 24-well plates coated with anti-CD3 (Biolegend) at a concentration of 2 µg/ml. Antibodies and cytokines were added at the time of plating at the following concentrations: 20 ng/ml IL-1b (Peprotech), 40 ng/ml IL-6 (Peprotech), 5 µg/ml anti-IL-4, (eBioscience), 10 µg/ml IFNγ (BD bioscience), 5 µg/ml anti-CD28 (Biolegend). On day 3 of polarization, 10 U/ml IL-2 (Roche) and 20 ng/ml IL23 (R&D Systems) were added. Polarized Th1 and Th17 cells were used on day 7 of polarization to generate immunocompetent skin constructs.

### Quantitative real-time PCR

Human naive CD4 +T cells were polarized to Th1 and Th17 as described above. RNA was extracted using the RNeasy Mini kit (Qiagen, Valencia, CA). The RNA was then DNAase I treated (Qiagen) and cDNA was synthesized using SuperScript III (Invitrogen, Carlsbad, CA). Quantitative real-time PCR was then performed using GAPDH to normalize following the 22ΔΔCT method. The primer sequences used were: IFNγ, forward, 59-GCA TCG TTT TGG GTT CTC TTG GCT GTT ACT GC -39, reverse, 59-CTC CTT TTT CGC TTC CCT GTT TTA GCT GCT GG -39; IL4, forward, 59-CCT CTG TTC TTC CTG CTA GC -39, reverse, 59-CCG TTT CAG GAA TCG GAT CA; IL13, forward, 59-GGA AGC TTC TCC TCA ATC CTC TCC TGT T-39, reverse, 59-GCG GAT CCG TTG AAC CGT CCC TCG CGA AA-39; IL17A, forward, 59-ACC AAT CCC AAA AGG TCC TC-39, reverse, 59-GGG GAC AGA GTT CAT GTG GT-39; IL17F, forward, 59-TGA AGC TTG ACA TTG GCA TC-39, reverse, 59-TTC CTT GAG CAT TGA TGC AG-39; IL22, forward, 59-GCA GGC TTG ACA AGT CCA ACT-39, reverse, 59-GCC TCC TTA GCC AGC ATG AA-39.

### Flow cytometry

To analyze T cells that migrated into the skin constructs, skin constructs were incubated in 1 mg/ml collagenase type IV (Worthington) for 1 h, vortexing every 20 min. The cell suspension was filtered through a 70 μm cell strainer and washed with PBS with 2% FBS before staining. Isolated cells were stained using anti-CD3 FITC (OKT3, Biolegend), anti-CD25 PerCP 5.5 (M-A251, BD Bioscience), and anti-CD69 Pacific Blue (FN50, Biolegend).

To analyze intracellular cytokines, the cells were stimulated by adding cell stimulation cocktail (eBioscience) for 1 h in addition to protein transport inhibitor (BD Biosciences) for 4 h. Cells were fixed and permeabilized using the BD Cytofix/Cytoperm kit (BD Biosciences) according to the manufacturer’s instructions and were stained using anti-IFNγ PE (4 S.B3, BD Biosciences), anti-IL-17A BV421 (BL168, Biolegend). All stained cells were analyzed on a FACS LSR II (BD Biosciences) and FCS express 6.0 (De Novo Software).

### Isolation of CCR6+ or CLA+ T cells and CCR6−CLA- T cells using BD Influx™

Cells were labeled with anti-CD3 V500 (UCHT1, BD biosciences), anti-CCR6 PE-cy7 (11A9, BD Biosciences), and anti-CLA BV605 (HECA-452, BD Biosciences) and sorted using a BD Influx™ (BD Biosciences). Purity of sorted cells was greater than 95%.

### Enzyme-linked immunosorbent assay (ELISA)

Supernatants were collected on day 2 and day 4 after generation of immunocompetent skin. Using commercially available kits, IFNγ (Mabtech), IL-17A (Mabtech), TNFα (R&D systems), IL-1b (Mabtech), IL-6 (Mabtech), IL-8 (Mabtech),and CCL20 (R&D systems) were assayed as instructed by the manufacturers.

### Immunofluorescence staining

Immunofluorescence staining was performed in OCT-embedded frozen tissues. After dry for 2 hours in 37 °C, tissue sections were rinsed with PBS and non-specific binding was blocked using 1% BSA and 5% goat serum in PBS containing 0.1% Triton-X-100 for 60 minutes at RT. Samples were incubated with primary antibodies (Keratin 14, 1:1000, eBioscience, Keratin 16, 1:400, Abcam, PPP6c, hBD2, 1:400, Abcam, CD3, 1:40, eBioscience)) overnight at 4 °C. After appropriate washing with PBS, samples were incubated with fluorophore-conjugated secondary antibodies (Goat anti-Rabbit 594, 1:500, Invitrogen, Goat anti-Mouse 488, 1:500, Invitrogen) for 1 hour at RT. Slides were coverslipped using Vectashield mounting media containing 4′,6-diamidino-2-phenylindole (DAPI) (Vectashield).

Wholemount tissue staining was performed similarly to the staining of the sections except that the tissues were incubated in the primary antibody for 3 days and the secondary antibody for 2 days. The tissue was cleared by benzyl alcohol to benzyl benzoate mixture (1:2) before imaging. Samples were examined using a Zeiss LSM 5 Exciter confocal laser scanning microscope.

### Quantitative reverse transcription-polymerase chain reaction (RT-PCR)

Total RNA was extracted using the RNeasy Plus Mini Kit (Qiagen; Hilden, Germany), according to the manufacturer’s instructions. cDNA was synthesized from 1 μg of total RNA using the Transcriptor First Strand cDNA synthesis kit (Roche Applied Science; Mannheim, Germany). Quantitative RT-PCR was conducted in triplicate using TaqMan master mix (Applied Biosystems; Foster City, CA) and the Real-Time PCR System (Applied Biosystems). mRNA expression of *collagen I* (Hs00164004_m1), *collagen III* (Hs00164103_m1), and *TGF-β1* (Hs00998133_m1) were normalized to glyceraldehyde-3-phosphate dehydrogenase (*GAPDH*) (Hs02758991-g1). Relative quantification was performed using Applied Biosystems 7500 software v2.0.1.

### Statistical analyses

All assays were repeated at least in triplicate, and the data are presented as the mean ± standard deviation (SD). Results were analyzed using one-way ANOVA. Bonferroni test was used for post-hoc analysis. Deviations were considered statistically significant when *p* < 0.05.

## Supplementary information

Supplementary information

## Data Availability

Data supporting the findings of this study are within this manuscript or available from the corresponding authors upon reasonable request.
