# Supplementary material for: Recapitulating T cell infiltration in 3D psoriatic skin models for patient-specific drug testing
Source: Sci Rep. 2020 Mar 5;10:4123. doi: 10.1038/s41598-020-60275-0 (PMC7057979; doi:10.1038/s41598-020-60275-0)
Supplement: Supplementary file 1 — Supplementary information [file 41598_2020_60275_MOESM1_ESM.docx]

Recapitulating T cell infiltration in 3D psoriatic skin models for patient-specific drug testing

**Authors:** Jung U Shin^1,2†^, Hasan E. Abaci^1†^*, Lauren Herron^1^, Zongyou Guo^1^, Brigitte Sallee^1^, Alberto Pappalardo^1^, Joanna Jackow^1^, Eddy Hsi Chun Wang^1^, Yanne Doucet^1^ and Angela M. Christiano^1,3^*

**Supplementary Materials:**


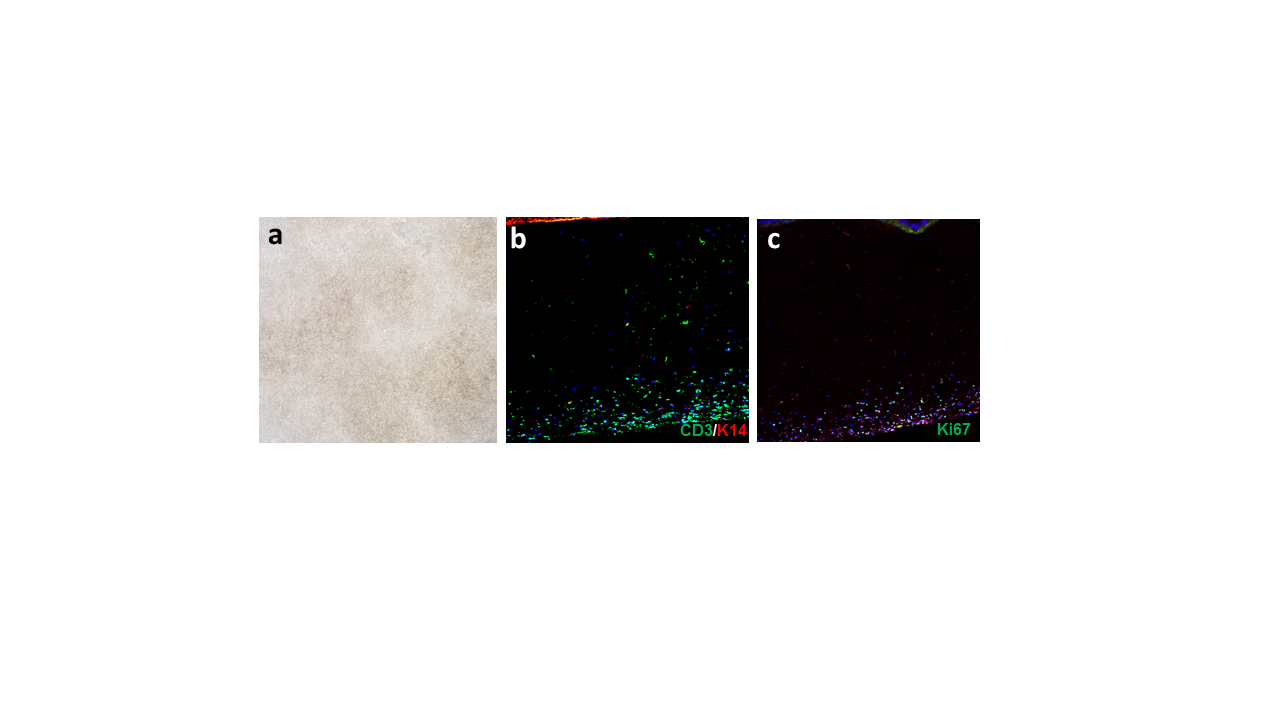


**Supplementary Figure 1. Attachment of T cells on acellular collagen gel layer and their migration into the skin.** **(a)** One day after attachment of T cells on acellular collagen gel examined under optical microscope. **(b)** Immunofluorescence staining of T cell-bearing HSCs with K14 (red) and CD3 (green) in the left panel, and **(c)** CD3 (red) and Ki67(green) in the right panel.


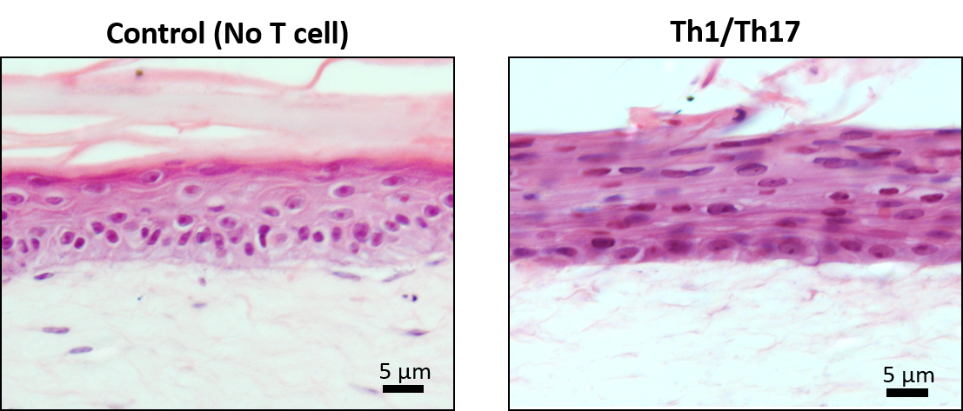


**Supplementary Figure 2. H&E staining of control HSCs (left panel) and HSCs with Th1 and Th17 cells after day 4 (right panel).**


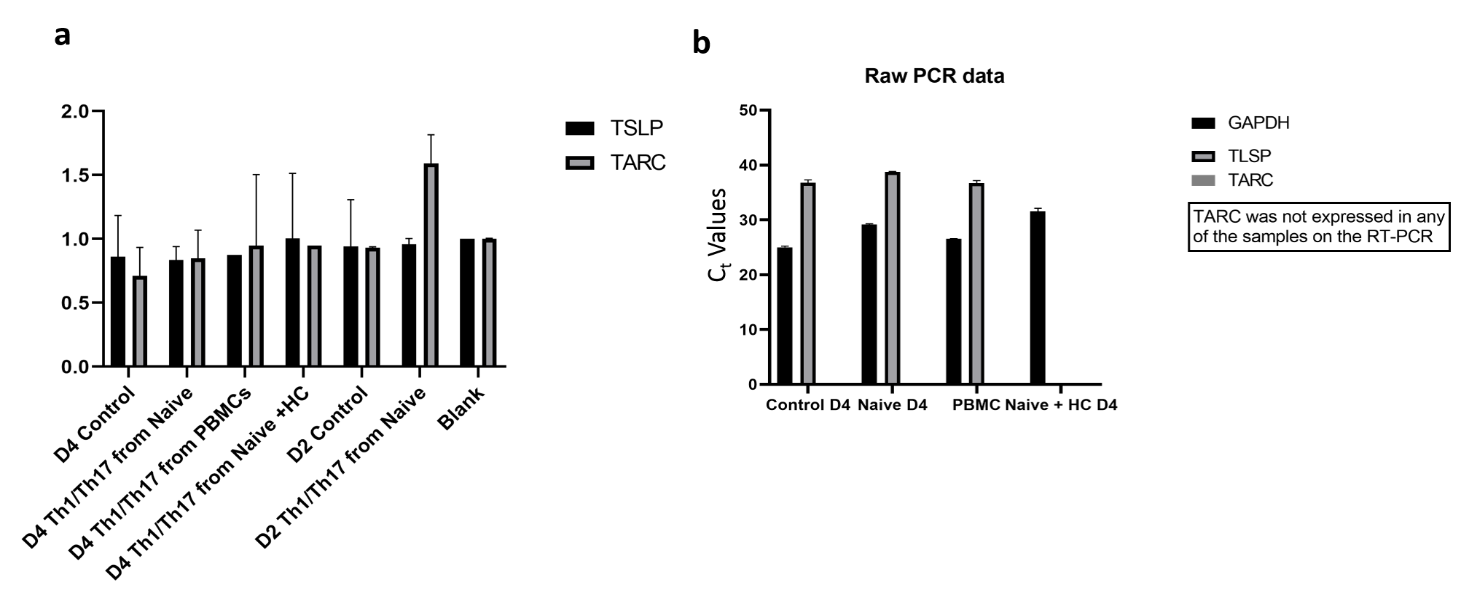


**Supplementary Figure 3. Gene expression and secretion of atopic dermatitis-related cytokines, TSLP and TARC, in pHSCs. (a)** ELISA data for TSLP and TARC obtained using media collected from control HSCs, untreated pHSCs, and hydrocortisone-treated pHSCs show no significant difference from the blank culture medium. **(b)** Raw Ct values of qRT-PCR performed for RNA collected from the epidermis of pHSCs show no expression of TARC and negligibly low expression of TSLP (> 35 cycles) for all conditions.


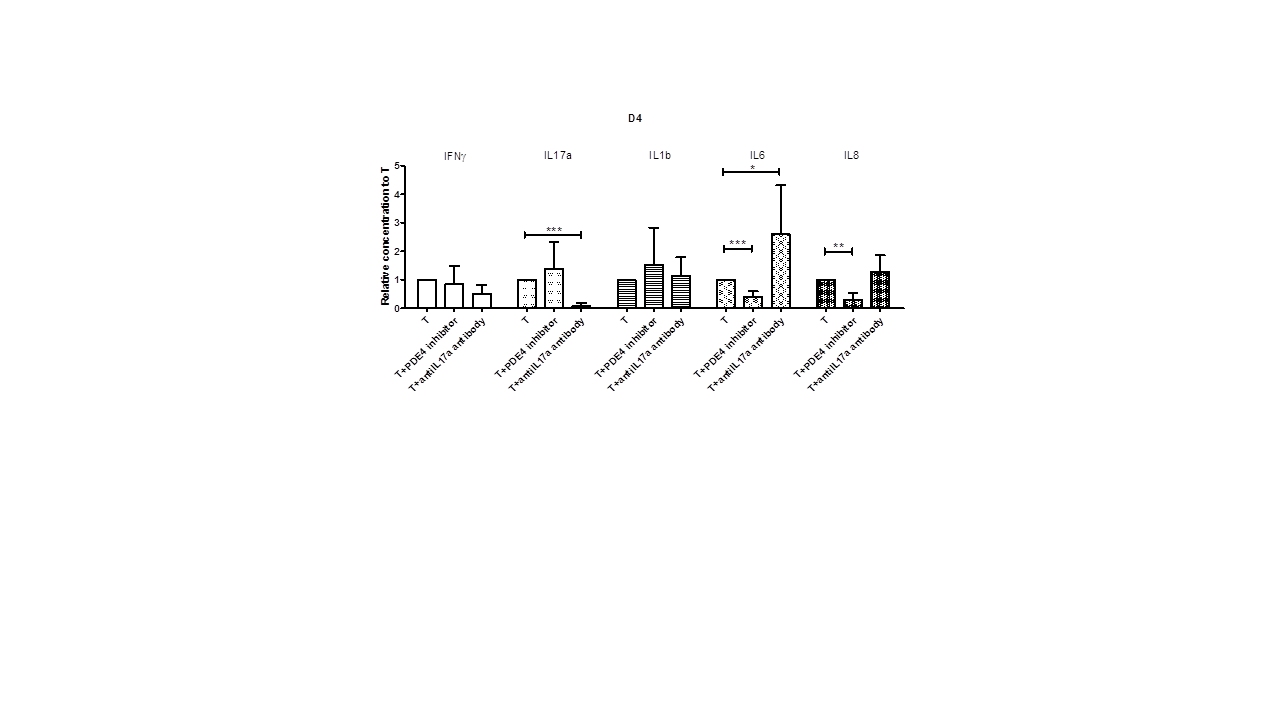


**Supplementary Figure 4. Effect of PDE4 inhibitor and anti-IL17a antibody on cytokine secretion in Th1/Th17-incorporated 3D skin.** Relative cytokine secretion of IFNγ, IL-17a, IL-1b, IL-6, and IL-8 from Th1/Th17-bearing HSCs after PDE4 inhibitor or anti-IL17a antibody treatment on day 4.
